# Supplementary material for: Content-rich biological network constructed by mining PubMed abstracts
Source: BMC Bioinformatics. 2004 Oct 8;5:147. doi: 10.1186/1471-2105-5-147 (PMC528731; doi:10.1186/1471-2105-5-147)
Supplement: Additional File 2 — The original results of the above study (non-essential files are deleted to keep the file size under the limit set by BMC bioinformatics). [file 1471-2105-5-147-S2.bz2 › chilibotAdditionalFile2/dip10/343ID8128231E4303/html/IL2RG_IL7.html]

 


 **IL2RG** and **IL7** 
  
Found 129 abstracts in PubMed, retrieved 10.  
 

 What does Google say? 
 PDF only 
| .edu only 

---

**Interactive relationship** (e.g. stimulation, inhibition, etc)

**Neutral relationship**- Previously it was known that the IL 2 and IL 7  [ **IL7** ]  receptors shared the common cytokine receptor gamma chain, gamma c  [ **IL2RG** ] , which suggested a possible competition between these cytokines for a receptor component.  Ref: 12354940 Proc Natl Acad Sci U S A, 2002
- Our laboratory has identified an X linked severe combined immunodeficiency XSCID  [ **IL2RG** ]  in dogs that is the result of mutations in the common gamma chain gammac subunit of the interleukin 2 IL 2, IL 4, IL 7  [ **IL7** ] , IL 9, IL 15, and IL 21 receptors.  Ref: 12857971 Immunol Res, 2003
- Interleukin IL 2, IL 4, IL 7  [ **IL7** ] , IL 9, IL 15, and IL 21 form a family of cytokines based on their sharing the common cytokine receptor gamma chain, gamma c  [ **IL2RG** ] , which is mutated in X linked severe combined immunodeficiency SCID.  Ref: 12435740 J Biol Chem, 2003

**Non-interactive relationship** (e.g. studied together, co-existance, homology, etc.)

- In addition to IL 7  [ **IL7** ] , several other cytokines that employ gamma c  [ **IL2RG** ]  as part of their receptor were also capable of stimulating D1 cell survival and proliferation.  Ref: 12609543 J Immunol Methods, 2003
- The common cytokine receptor gamma chain gamma c  [ **IL2RG** ] , an essential component of the receptors for IL 2, IL 4, IL 7  [ **IL7** ] , IL 9, and IL 15, is critical for the development and function of lymphocytes.  Ref: 12093291 Biochemistry, 2002
- To address whether IL 7  [ **IL7** ]  receptor R activity is essential for early B lineage development in vivo, we examined the frequencies of CLPs and downstream pre pro and pro B cells in adult mice lacking either the alpha chain or the common gamma chain gamma c  [ **IL2RG** ]  of the IL 7R.  Ref: 12208884 J Exp Med, 2002
- IL 21R shares the common gamma chain gamma c  [ **IL2RG** ]  with the receptors for IL 2, IL 4, IL 7  [ **IL7** ] , IL 9, and IL 15.  Ref: 12244150 J Immunol, 2002
- The receptor consists of two chains IL 7  [ **IL7** ]  specific alpha chain CD127 and the common gamma c  [ **IL2RG** ]  chain CD132  [ **IL2RG** ]  which is a component of several other cytokine receptors IL 2, IL 4, IL 9 and IL 15.  Ref: 12644318 Immunol Lett, 2003
